# A Link is not Enough – Reproducibility of Data

**DOI:** 10.1007/s13222-019-00317-8

**Published:** 2019-06-13

**Authors:** Mateusz Pawlik, Thomas Hütter, Daniel Kocher, Willi Mann, Nikolaus Augsten

**Affiliations:** 1 Database Research Group, University of Salzburg, Salzburg, Austria; 2 Celonis SE, Munich, Germany

**Keywords:** Data preparation process, Reproducibility

## Abstract

Although many works in the database community use open data in their experimental evaluation, repeating the empirical results of previous works remains a challenge. This holds true even if the source code or binaries of the tested algorithms are available. In this paper, we argue that providing access to the raw, original datasets is not enough. Real-world datasets are rarely processed without modification. Instead, the data is adapted to the needs of the experimental evaluation in the data preparation process. We showcase that the details of the data preparation process matter and subtle differences during data conversion can have a large impact on the outcome of runtime results. We introduce a data reproducibility model, identify three levels of data reproducibility, report about our own experience, and exemplify our best practices.

## Background on Reproducibility

Reproducibility is essential to scientific research. When new algorithms are proposed, they must be compared to existing work. Often, this process is frustrating due to missing information. First, an implementation of the competitors’ approach is required. Missing details in the pseudocode, uncovered corner cases that are not discussed in the paper, and the lack of source code often make it cumbersome to reimplement existing work. Second, to be able to repeat previous experimental results, the datasets for these experiments are also required. Being able to repeat previous experiments is essential to verify one’s own implementation of the competing approaches and to explain diverging results, runtime improvements, or other experimental outcomes. In this paper, we focus on the reproducibility of data, in particular on the data preparation process, which has received little attention. We introduce *RPI*, a data reproducibility model that consists of three elements: raw data, preparation instructions, and input data. Based on our model, we devise three data reproducibility levels (not replicable, replicable, and reproducible) and point out the importance of a well-defined data preparation process. For this work, we adapt the experiments reproducibility terms defined by the ACM [1].

*Replicability.* The original input data is given, and the experiments can be executed by another research group verifying the published results.

*Reproducibility.* Additionally to replicability, the experiments can be evaluated on new data configurations.

A third term used by ACM is *repeatability*, which requires only the original authors to repeat their own results. The requirements on data are identical to replicability.

**Table 1:** Summary of reproducible papers (ACM SIGMOD and PVLDB)

Conference	# Accepted	# Listed as reproducible	Reproducibility ratio	# Artifacts currently available
ACM SIGMOD 2015	124	10	8.06%	9
ACM SIGMOD 2016	137	14	10.22%	13
ACM SIGMOD 2017	105	8	7.62%	6
ACM SIGMOD 2018	107	8	7.48%	7
PVLDB 2018	129	6	4.65%	6
	**602**	**46**	**7.64%**	**41**

### Reproducibility in the database community

A number of efforts in the database community aim to raise the awareness for the importance of reproducibility of experimental results. ACM SIGMOD [26] (since 2015) and PVLDB [23] (since 2018) encourage the authors to make their works reproducible and to share their research artifacts (source code, datasets, data preparation instructions). The reproducibility programs are organized by different committees, but are now developed in coordination since 2018. Besides the inherent benefits of higher expected impact and more recognition of the work, especially ACM SIGMOD provides additional incentives like monetary prices, badges visible in the ACM Digital Library (ACM DL), and a dedicated award: the ACM SIGMOD Most Reproducible Paper Award. The committees provide clear guidelines for a good reproducibility package, including system installation, execution of all experiments, and recompilation of the original publication with new plots.

Despite these efforts, the number of reproducible papers in the database community is low. We verified the availability of the research artifacts for all papers listed as reproducible by ACM SIGMOD [26] and PVLDB [23]. First, we checked the availability of the source material in the ACM DL. If nothing was found in the ACM DL, we searched in the papers for links to source material (keywords: http, online, available, code, benchmark, data). Whenever we found a working link to the source material in the PDF or in the ACM DL, we consider the artifacts to be available (no extensive web search). We summarize our findings in Table 1. Sadly, only around 8% of all papers published at the corresponding venues are listed as reproducible. On the positive side, we notice that for most of those papers, the research artifacts are still available.

From a data perspective, the ACM SIGMOD and PVLDB guidelines only recommend to generate or fetch all needed input data without any further specifications [26]. This, however, is not enough. In subsequent sections, we demonstrate that data reproducibility is more complex and discuss various aspects that must be considered when providing data for reproducibility purposes.

### Related work

A recent extensive study [35] investigated 601 papers from the top computer science outlets, eight conferences and five journals (including ACM SIGMOD’12, PVLDB’12, and ACM TODS’12 from the database community). The study focused on building and executing the source code. The authors put also much effort into obtaining the source code, when the paper did not point to it (that included contacting authors and institutions). Data reproducibility, which is the focus of this paper, is not further investigated. The study, among other insights, reveals that out of 601 verified papers (216 in DB outlets), 402 are backed up by source code (DB: 145), for 130 papers the source code builds without problems (DB: 54), and in 85 cases the code location is given in the paper (DB: 14).

An interesting viewpoint is presented by Thomas Neumann [30]: Empirical evaluation is essential and provides much insight. However, the experiments hurt the review process. Intentionally or not, authors present results to please the reviewers, and the reviewers expect only positive results. This holds true also for the selection or creation of datasets, which sometimes are tailored to show the superiority of a specific algorithm. In this paper, we argue for disclosing all steps of the data preparation process that were applied to the raw data (in the case of real-world datasets) to discourage the fine-tuning of experimental data to specific algorithms.

A number of related works treat different aspects of reproducibility. Currim et al. [36] show how to correctly measure runtime. Vogelsgesang et al. [47] discuss the properties a benchmark should have to mimic real-world workloads. The Koblenz Network Collection [16] is an example of a well-documented collection of datasets in the domain of graphs. For Ludwig and Geyer [43], optimal reproducibility means that the results of individual program runs are bitwise reproducible. From this perspective, the authors discuss trade-offs (e.g., forced determinism may lead to less efficient implementations), uncertainties (e.g., compiler optimizations, libraries), and reproducibility on future hardware (e.g., Exascale, FPGAs/GPUs, and bit error probability for large main memory). This recent publication also highlights the importance of reproducibility in any computer-aided research area. The role of data reproducibility is not explicitly discussed, but we consider this as a requirement for bitwise reproducibility.

### Open Science

The Open Science movement advocates freely-accessible research. An essential part of Open Science is the availability of data and research artifacts, which is often required by research funding agencies. We looked into the guidelines of three national funding agencies in German speaking countries, the Austrian Science Fund (FWF) [14], the German Research Fund (DFG) [7], and the Swiss National Science Fund (SNSF) [27]. They agree that research data should be publicly and freely available. They propose to use archiving services, e.g., Zenodo [29] (a collaboration of CERN [2] and OpenAIRE [22]), and the DRYAD Digital Repository [9], or online data repository indexes such as the Registry of Research Data Repositories (re3data) [24]. Other data repositories and online indexes include EUDAT [12], Harvard Dataverse [15], figshare [13], and Mendeley Data [5].

Publishers support Open Science by offering hosting of research artifacts together with the corresponding publications, e.g., ACM SIGMOD publishes the artifacts in ACM DL [26], and Elsevier offers a dedicated Data in Brief [4] journal.

Governments join Open Science by hosting publicly-available datasets that can be further used for research. Examples include the data portals of the European Union [11], Austria [18], Germany [19], Switzerland [20], and the United States [21].

*Outlook.* The paper is organized as follows. We motivate the discussion on data reproducibility based on our experiences with tree structured data in Section 2. In Section 3, we introduce our RPI data reproducibility model and define each of the RPI elements. Section 4 discusses the situations when the definitions of RPI elements are not satisfied. We map subsets of RPI elements to one of three reproducibility levels in Section 5. The discussion on availability aspects of the RPI elements is orthogonal and examined in Section 6. We analyze the reproducibility levels of our related work in similarity joins in Section 7 before we exemplify our best practices on data reproducibility in Section 8 based on two of our recent works. We conclude and summarize in Section 9.

**Table 2:** Differences in XML-to-tree conversions

	Tree size			Label	Join result	Time
	avg	max	min	# diff.	# pairs	[s]
A	26.1	2987	9	18.5$\cdot 10^{6}$	167365	142
B	21.5	1501	3	14.5$\cdot 10^{6}$	12446240	326

**Fig. 1:**
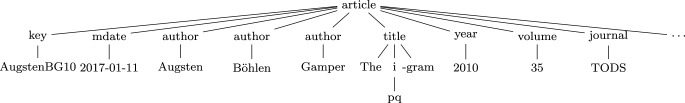
XML tree using *Conversion A*

**Fig. 2:**
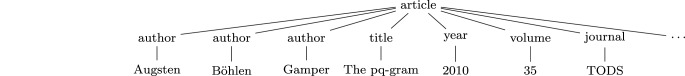
XML tree using *Conversion B*

## A Link is not Enough

A common practice, when describing experimental data, is to provide some data statistics and a link to the data source. This, however, is not enough. In this section, we showcase how missing details in the description of the data preparation process may affect the experimental results. We motivate the discussion with our experience gained while working on similarity join algorithms for sets [44] and trees [38]. As part of the experimental evaluation in these works, we also compared to previous solutions. For both publications, we often had to deal with unavailable source code and datasets that were not reproducible. As a result, a disproportionately large share of the overall effort was to implement and test related works. Moreover, comparing the experimental results was almost impossible, although some datasets were used in multiple papers. One of those datasets is DBLP [6] which we use to illustrate our example.

DBLP [6] stores bibliographic data in XML format and includes, among others, authors, titles, and venues of computer science publications. Due to its availability and intuitiveness, the DBLP dataset has been used in many works for experimental purposes, e.g., as a collection of sets [44, 45], as a collection of trees [37, 38, 46], as a large hierarchical document [34, 40], and as a coauthor network graph [42, 49]. In this section, we show the impact of differences in the data preparation process that converts raw DBLP XML data into the desired input format.

In our case of tree similarity joins, each article in DBLP is represented as a tree. Unfortunately, there are various ways to represent XML as a tree. We consider the following XML snippet showing one article from DBLP.

 
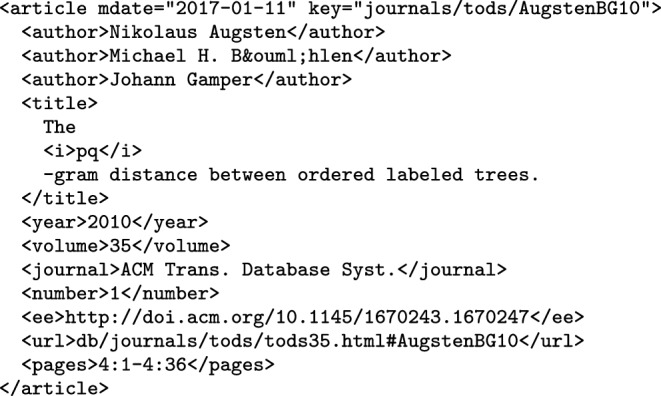


This snippet contains element tags (pairs of the form <tag_name></tag_name> that can be nested), attributes (key-value pairs of the form key=“value”), and text values surrounded by tags (e.g., Nikolaus Augsten surrounded by the <author> tag). While it is clear that the structure of the XML document encodes hierarchical dependencies between data items, there are many possibilities to get from XML to a tree. Figure 1 shows one of them (we abbreviate the text values and use only selected element tags for the visualization). This representation considers all element tags and attributes. Note that the title is split into four nodes due to the <i> HTML tag (represents text in italics). Other options include, for example, discarding all attributes, removing HTML tags from titles, discarding tag names, combining tag names with their text values, or discarding text values of elements. Similar decisions must be made when converting XML snippets to sets, e.g., which tags to consider, should tag names be set elements, is the title a single element or is it tokenized, on what characters should strings be tokenized.

We now take a look at a specific example in which we convert the DBLP dataset into trees using two different approaches and perform a tree similarity join [38]. *Conversion A* is used in our data preparation process [38] and *Conversion B* is used by Tang et al. [46]^1^. *Conversion A* is illustrated in Figure 1. All XML elements are preserved, including tag names and their text values, attribute keys and values. Attributes are placed as first children of a tag they belong to. Attribute key-value pairs are in a parent-child relationship. *Conversion B* is illustrated in Figure 2. In contrast to *Conversion A*, all attributes are discarded and HTML tags are removed from titles such that each title text value is a single node.

Table 2 shows dataset statistics for *Conversions A* and *B* including tree sizes, the number of different labels in a collection, the number of similar tree pairs in the result set, and the corresponding join runtimes^2^.

The similarity join on dataset *B* runs twice as long as the same join on dataset *A*. More significantly, the number of result pairs differs by two orders of magnitude. Clearly, if no common conversion strategy is used, the results of different works are not comparable and the difference in the results may be large. In our experience, the description of the conversion step is often omitted or lacks relevant details. Motivated by this experience, in the following sections, we propose a data reproducibility model and identify the key requirements to make data reproducible.

## The RPI Data Reproducibility Model

**Fig. 3:**
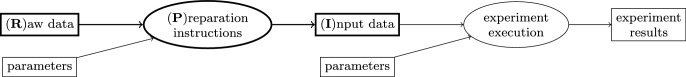
RPI data reproducibility model

Previous reproducibility studies focused on experiments assuming that the input data is given. However, the input data is usually obtained through a complex process. We focus on the data preparation process and present our RPI data reproducibility model (cf. Figure 3). We identify three elements, which we call *RPI elements*. The *raw data* (R) is converted into experimental *input data* (I) using *preparation instructions* (P).

*(R)aw data* is a snapshot of the dataset, in the original format, from which the experimental data is created, for example, the XML snapshot of the DBLP dataset on Nov. 1, 2017. Raw data is usually further processed and converted into the desired input format.

*(P)reparation instructions* are a complete and unambiguous sequence of instructions that specify how raw data is transformed to the input data for the experimental evaluation. They may be given in text form (typically as part of a publication), or ideally as an automated script. The advantage of a script is that it is less prone to missing details or misunderstandings. In some cases the raw data is identical to the input data, then no preparation instructions are needed.

*(I)nput data* is an exact copy of the data in terms of both content and format that was used as an input for the experiment. No further modifications are needed to execute the experiment. For example, in our scenario of tree similarity joins, the input data is a text file with one tree per line in a so-called bracket notation, and the trees are sorted by size (in case of ties, sorting bracket notation strings lexicographically).

Unfortunately, in our experience, research papers are rarely supported with artifacts satisfying our definitions of RPI elements. Next, we identify such situations and discuss their implications on data reproducibility.

## Satisfying Definitions of RPI Elements

The RPI elements are at the core of data reproducibility. Our definition of the RPI elements ensures that the data artifacts are identical to the original data and the entire process of data preparation is reproducible. As an outcome, the experimental results can be reproduced, and additionally all information to modify and apply the process to new data is at hand. For each RPI element, we address circumstances when its definition is not satisfied.

### (R)aw data

The raw data primarily serves as a source for the input data. Therefore, it is often a superset of the input data. Providing the raw data is useful to put the input data into a larger context. Furthermore, we learn which parts of the data were discarded, or we may use the raw data to generate new inputs.

Publishing only a link to the data source is not enough. The host may change and the data will no longer be available under the old URL. Further, the data may evolve, which may cause differences in the experimental results.

In some cases, the input data is identical to the raw data, e.g., when customized synthetic data are generated. In this case, we consider both raw (R) and input data (I) as given.

### (P)reparation instructions

Preparation instructions should document the process of getting from the raw data to the input data. Giving only an intuition is not enough since such processes may be difficult to repeat, e.g., third-party tools are not compatible or not available anymore. Ideally, a conversion script that automates the process from downloading the raw data snapshot to producing the experimental input data in a desired format is provided. If any information is missing or incorrect, the data preparation process cannot be repeated.

Providing the preparation instructions is not always possible. For example, if datasets are inherited from previous publications, the details about the preparation instructions or the original source of the data may not be recoverable.

### (I)nput data

To observe the same results as in the original experiments, the input datasets must be identical. If no snapshot of the input data is provided and the underlying source changes, we are not able to recover the original input data. Providing only the input data hides the data preparation process and the details of extracted information, and the transformation to the input format remains unclear. In the case of synthetic input data, especially when random values are involved, it has to be ensured that the generation process is deterministic.

In order to achieve replicability or reproducibility of data, we consider our RPI model to be binary, i.e., the RPI elements must be given and satisfy our definitions. We observe that a common practice is to provide only a link to raw data or data statistics. Such information may help but does not guarantee that the datasets are identical. To allow data verification, a checksum could be provided additionally to the data.

Depending on which RPI elements are provided, data exhibit different degrees of reproducibility, which we define in the next section.

## Data Reproducibility Levels

**Fig. 4:**
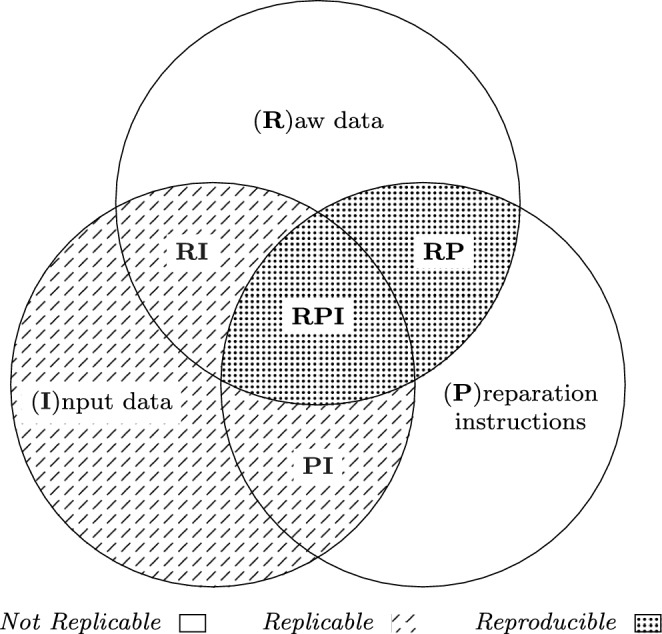
Data reproducibility levels

Reproducibility of experiments and measurements is a goal in various research communities. According to the ACM, an experiment is *replicable* if the same result can be obtained using the author’s own artifacts, and *reproducible* if the same result can be obtained using independently developed artifacts [1]. We adapt these definitions to data. We place the RPI elements in a Venn diagram (cf. Figure 4), where each region represents one of three data reproducibility levels:

- *Not replicable (no pattern).* Input data cannot be reproduced. Thus, the experiments cannot be executed.
- *Replicable (lines).* Original input data is provided. The experiments can be executed on the same input and are expected to lead to the same results.
- *Reproducible (dots).* Experiments can be executed on the original input data, and new data configurations and datasets are easy to develop.

### Not replicable: P, R

The input data, which is required to repeat an experiment, cannot be generated if only (R) or (P) is given. The raw data (R) is a superset of the input data in a different format. Preparation instructions (P) require raw data as an input.

### Replicable: I, RI, PI

This level guarantees that the experiments can be executed on the original input, such that the results can be verified.

Input data only (I) allows to repeat the experiments and verify the original results. Since the data preparation process is not known, it is difficult to extend the experiments with new datasets or vary the parameters of the preparation instructions, for example, varying the length of n‑grams when tokenizing string data for set similarity queries.

If the data preparation process is not documented (RI), but both raw and input data are given, the preparation process can potentially be reverse-engineered, which is not possible in the case of (I). However, this is typically a cumbersome process that is not always successful. For the rest, (RI) and (I) have similar issues.

When a snapshot of raw data is not available (PI), we gain a good understanding of the data preparation process, but some details may remain unclear. For example, some relevant parts of the raw data may not have been included and alternative approaches to prepare the data cannot be considered.

### Reproducible (RPI, RP)

Ideally, raw data, input data, and preparation instructions (RPI) are present. Then, we can repeat the experiments and further extend them with different parameter configurations and new datasets. If only the input data is missing (RP), based on our definitions (cf. Section 3), we can replicate it by applying the preparation instruction on the raw data snapshot. Note, however, that the data preparation process may be very expensive, require specific hardware or settings that are different from the experimental settings in which the resulting input data is used. If the input data is given, it can also serve as a correctness test for the preparation instructions.

The guidelines provided by the committee of the ACM SIGMOD Reproducibility program [26] are equivalent to the (I) element of the RPI model. Hence, the guidelines only ensure replicability of data.

## Data Availability

In order to achieve data reproducibility, the relevant RPI elements must be made available to other research groups. We consider availability an orthogonal property and discuss it separate from the RPI model in this section.

Ideally, a research paper contains all information on how to access the experimental data, the information persists, and remains valid over time. Unfortunately, from our experience, this is often not the case. We study typical obstacles in data publishing and observe two aspects, legal and technical, that may be at the source of unavailable data.

### Legal Aspect

Before data is published, legal aspects must be taken into consideration. For example, publishing data may cause economic risks for the data owner, violate license agreements or privacy laws. We distinguish public, proprietary, and sensitive data.

*Public data* contains information, whose disclosure does not cause any risk, e.g., DBLP bibliographic data, IMDB movies database, Swissprot proteins, open government data, or automatically-generated synthetic data. Although public datasets are available online, they often come with licenses that protect the contribution of the authors or the data itself. As with source code licensing, a number of license schemes have been proposed for data, e.g., the licenses by Creative Commons [3] or Open Data Commons [17]. A good source of information about licenses for research data is the guide by Alex Ball from Digital Curation Centre [31].

In some cases, however, data cannot be made available. *Proprietary data* often comes from industry. Disclosure of such data may put a company at legal or financial risk. *Sensitive data*, for example, data from medical applications, contains personal information, and such data cannot be legally published. Unfortunately, in both cases, data reproducibility is usually not achievable. When data cannot be published due to legal reasons, the experiments performed with these datasets cannot be verified by independent research groups. We argue that the core experimental results in a paper should not be based solely on unpublished data, but should be supported also by datasets that are accessible to other researchers. An interesting model to deal with legal issues is to make the experimental data available only to individual research groups that sign a non-disclosure agreement and verify the experimental results.

### Technical Aspect

When the legal issues are solved, a dataset may be shared with other researchers. We consider data to be available if it can be accessed directly on the Web, or indirectly by contacting the data owner. A common practice is to host the datasets. We identify the following technical challenges: hardware resources, software compatibility, financial resources, and continuous availability.

*Hardware resources.* Experimental data may be large. To host it, dedicated disk space and possibly a server machine are required, which may not be available. In such cases, archiving services can be used, e.g., Zenodo [29] or the DRYAD Digital Repository [9], recommended by Austrian, German, and Swiss science funds.

*Software compatibility.* Often, third-party software tools are used in the data preparation process, e.g., to convert or generate data. Such tools, as well as programming languages, evolve over time, and may lead to compatibility issues. A periodic execution of the preparation instructions may identify such situations. In order to avoid unsupported software, compatible versions can be specified, and if these versions are no longer available, updating the data preparation process may be necessary. A commonly used technique to provide software applications are container systems like Docker [8]. Still, the problem with third-party software, which is no longer supported, remains. If licenses allow, the required versions of the software tools may be shipped together with the data.

*Financial resources.* In some cases, the collection, curation, and publication of data may require additional financial resources, e.g., fee-based datasets or new hardware requirements to process and store large datasets. Research funding agencies, e.g., FWF [14], DFG [7], SNSF [27], allow the applicants to request additional funds for preparing, archiving, and publishing research data.

*Continuous availability.* Research articles are cited often many years after publication. Therefore, research data is ideally available forever. However, it is common for researchers to change their affiliation. Maintaining data under such circumstances is challenging. Possible solutions include publishing data directly with the corresponding article on the publisher’s website (e.g., ACM SIGMOD Reproducibility [26] or Elsevier’s Data in Brief [4]), and providing standardized and permanent access to data through a persistent identifier (archiving services, e.g., Zenodo [29], DRYAD [9], and indexes, e.g., re3data [24], Mendeley Data [5]).

## Data Reproducibility in Similarity Joins

We now go back to our use case, and review the data reproducibility levels of five related works on tree similarity joins [38] and five works on set similarity joins [44].

### Set similarity joins

Two publications provide the input data (I) and, therefore, are replicable. In particular, for [48] the input data is published online, and for [33] we got the data directly from the authors. Bayardo et al. [32] provides preparation instructions (P) for one out of three datasets. According to the main author, the other datasets are proprietary. In the case of [45], the raw data (R) was provided by the authors. However, the input data could not be generated due to incomplete preparation instructions. According to the main author, the algorithm was implemented in a larger framework which could not be published as a whole at that time. In the case of [50], we accessed the raw data (R) on the corresponding project website in 2016, but it was no longer available at the time of writing this paper.

### Tree similarity joins

For all related works [37, 39, 41, 46, 51], in the best case, links to raw data without timestamps are provided with additional statistics; in the worst case, only the name of the dataset is mentioned. This, however, does not satisfy the definition of the R element. Hence, the datasets are neither replicable nor reproducible. For the most recent publication [46], we contacted the authors, but they could not provide any of the datasets used in their paper. Therefore, we applied our own data preparation process to the raw datasets (which we downloaded following the links provided in the paper). We experimented with different settings to prepare the data, but for none of the preparation variants, the statistics of our output matched the statistics reported by the authors. Possible reasons are that the raw data has changed over time or different data preparation instructions have been used by the original authors.

Summarizing, three out of ten papers reach the level of data replicability; none of the papers satisfies our definition of data reproducibility.

## Best Practice Example

Two recent research experiences [38, 44] required us to compare to a number of related works. As detailed in Section 7, in most cases the data was not even replicable. Motivated by this cumbersome experience, we decided to collect and publish all datasets used in our experimental evaluations. Our goal was to enable other researchers to easily reproduce our experiments. We assembled two collections of real-world datasets; one containing trees [28] and one containing sets [25].

### Set datasets

We provide at least the input data (I) and, based on the information gathered from the original authors, we add also the raw data (R) and the preparation instructions (P). Hence, replicability is ensured for all datasets.

### Tree datasets

We realized while writing this paper, that not all of our data were reproducible due to the absence of raw data snapshots. The licenses allowed us to publish the snapshots of the original raw data (R) for trees. Together with fully automated scripts (P), we achieve reproducibility for those datasets.

In addition to the data used in our experiments, we also published the source code of our algorithms and the experimental framework [10, 28].

## Conclusion

In this paper, we highlight the importance as well as the challenges of data reproducibility. We discuss previous attempts by individual researchers, the database community, and funding agencies to raise the awareness for data reproducibility. We introduce RPI, a novel data reproducibility model that captures the data preparation process from raw data (R) through preparation instructions (P) to the input data (I) used in the experimental evaluation. Based on RPI, we define three reproducibility levels for experimental data: not replicable, replicable, and reproducible. We discuss technical and legal obstacles of data availability and propose how to overcome them.

With this paper, we hope to trigger a discussion in the database community and encourage a culture of data reproducibility.

## Footnotes

1 Since the authors could not provide the data, we devise the conversion from the example in their paper.

2 We use a distance threshold of 4. Please refer to [38] for more details.
